# Design, Synthesis and Crystal Structure of a Novel Fluorescence Probe for Zn^2+^ Based on Pyrano[3,2-c] Carbazole

**DOI:** 10.3390/molecules29225454

**Published:** 2024-11-19

**Authors:** Ziyin Xie, Qingwen Fang, Shuzhen Xiao, Jie Wang, Ping Lin, Chunmei Guo, Huihua Cao, Zhongping Yin, Lihong Dong, Dayong Peng

**Affiliations:** 1 College of Chemistry & Materials, Key Laboratory of Chemical Utilization of Plant Resources of Nanchang Jiangxi Provincial Key Laboratory of Improved Variety Breeding and Efficient Utilization of Native Tree Species, Jiangxi Agricultural University, Nanchang 330045, China; xieziyin2001@163.com (Z.X.); xiao888612@163.com (S.X.); wangjie951127@163.com (J.W.); 2 College of Forestry, Jiangxi Provincial Key Laboratory of Improved Variety Breeding and Efficient Utilization of Native Tree Species, East China Woody Fragrance and Flavor Engineering Research Center of National, Forestry and Grassland Administration, Jiangxi Agricultural University, Nanchang 330045, China; fqw912029833@126.com; 3 Jiangxi Key Laboratory of Natural Products and Functional Food, Jiangxi Agricultural University, Nanchang 330045, China; linping0322@henu.edu.cn; 4 Jiangxi Institute for Drug Control, Nanchang 330029, China; xiaobaitt_17@126.com; 5 Jiangxi Provincial Key Laboratory of Tea Planting and High Value Utilization of Characteristic Fruit Trees, Jiangxi Institute of Cash Crops, Nanchang 330013, China; hhcaojxjz@163.com; 6 College of Computer and Information Engineering, Jiangxi Agricultural University, Nanchang 330045, China

**Keywords:** fluorescence probe, Zn^2+^, pyrano[3,2-c] carbazole, crystal structure

## Abstract

Zinc is a trace element, which plays an important role in many biological processes. The deficiency of zinc will lead to many diseases. Thus, it is of great significance to develop fast and efficient quantitative detection technology for zinc ions. In this study, a novel fluorescence probe **FP2** was designed for Zn^2+^ quantification based on pyrano[3,2-c] carbazole. The structure of **FP2** was characterized by ^1^HNMR, ^13^CNMR, HRMS, and X-ray diffraction. In the HEPES buffer solution, **FP2** is responsive to Zn^2+^ and greatly enhanced. The pH value and reaction time were investigated, and the optimum reaction conditions were determined as follows: the pH was 7~9 and the reaction time was longer than 24 min. Under the optimized conditions, the concentration of **FP2** and Zn^2+^ showed a good linear relationship in the range of 0~10 μM, and the LOD was 0.0065 μmol/L. In addition, through the ^1^H NMR titration experiment, density functional theory calculation, and the job plot of **FP2** with Zn^2+^ in the HEPES buffer solution, the binding mode of **FP2** and Zn^2+^ was explained. Finally, the method of flame atomic absorption spectrometry (FAAS) and **FP2** were used to detect the content of Zn^2+^ in the water extract of tea. The results showed that the **FP2** method is more accurate than the FAAS method, which shows that the method described in this work could be used to detect the content of Zn^2+^ in practical samples and verify the practicability of this method.

## 1. Introduction

With societal development, increasing attention has been given to food safety. Zinc is the second most essential trace element in the human body after iron, which mainly exists in the form of Zn^2+^ in our body. It is of great importance in the regulation of various physiological responses [1]. Zn^2+^ is a key structural component of a large number of proteins, and it is involved in many biochemical processes in vivo, such as gene expression, apoptosis, enzyme regulation, nerve transmission, protein synthesis, etc. [2,3,4,5]. In addition, zinc ions will also affect certain cells in our body, such as antioxidants, memory, and immunity [6]. However, zinc Imbalance in zinc ion content can cause significant harm to the human body. Studies have shown that Zn^2+^ deficiency will lead to Type 2 Diabetes, Haemorrhagic Stroke, and Cardiovascular Disease, etc. Zinc deficiency can also increase the risks of allergic diseases and prostate diseases [7,8,9,10]. Excessive zinc ions can interfere with the absorption of trace elements in the human body, which may cause problems such as anemia, skeletal deformities, and decreased immune function [11]. Thus, it is of great significance to develop fast and efficient quantitative detection technology for zinc ions.

At present, the main analytical methods of Zn^2+^ are optical methods [12,13,14,15], inductively coupled plasma atomic emission spectrometry [16,17,18,19], inductively coupled plasma mass spectrometry [20,21,22,23], and atomic absorption spectrometry [24,25,26], etc. However, these methods face challenges such as complex operation, high cost, high detection limit, and low sensitivity. In recent years, the fluorescent probe detection method has been invented. This method has overcome most of the shortcomings in traditional detection techniques and has been widely used for the analysis and detection of metal ions [27]. Fluorescence probe technology has developed rapidly, and many research results have been obtained, including pyridine derivatives [28,29,30,31], quinoline derivatives [32,33,34,35,36,37], coumarin derivatives [30,38,39,40,41,42,43,44], etc. The development of organic heterocyclic chemistry has promoted the rapid development of fluorescent probe technology, and a wide variety of organic heterocyclic compounds have also provided a large number of raw materials for the development of fluorescent probe technology.

Carbazole is an aromatic nitrogen-containing heterocyclic compound composed of two benzene rings and a pyrrole ring. It has a double symmetry axis, excellent hole transport capacity, and rigid plane structure, and is usually used as an electron donor molecule. Its derivatives are widely used in organic light-emitting diodes and fluorescence sensors because of their easy availability of raw materials, good thermal stability, easy modification of structure, and high luminescence [45,46,47,48,49,50]. The clear coordination mode of probe receptors is crucial for host-guest chemistry. Therefore, selecting a receptor that can specifically bind to Zn^2+^is important for improving selective detection. Bis (pyridin-2-yl methyl) amine (DPA) is a compound used as a bidentate ligand in coordination chemistry. It contains three nitrogen atoms that provide lone pair electrons for chelation with metal ions, especially exhibiting good selective recognition ability for Zn^2+^. In recent years, due to its superior stability, specific recognition, low toxicity, and water solubility, DPA-based fluorescent molecular probes have made a leap forward in development [51,52,53].

Based on the excellent physical, chemical, and optical properties of carbazole derivatives, in this work, a novel fluorescent molecular probe, **FP2**, with strong blue fluorescence and coumarin as chromophore, was designed and synthesized from 4-hydroxycarbazole, Ethyl 4-chloroacetoacetate, and DPA as raw materials. The probe was used to quantitatively detect the content of Zn^2+^. The test results showed that the fluorescence probe has great potential for simple and sensitive detection of Zn^2+^.

## 2. Experimental

### 2.1. Materials and Reagent

All chemicals were purchased from Bide Pharmatech Ltd. (Shanghai, China) and Energy Chemical (Anqing, China, used without further purification). All the aqueous solutions needed for the experiment were prepared from ultrapure water obtained from the Milli-Q system. The tea was purchased from a local supermarket.

### 2.2. Instruments

The fluorescence spectra were measured on Cary Eclipse (VARIAN medical systems Palo Alto, CA, USA), the ultraviolet spectrophotometer was a UV-5200PC (Shanghai Aucy Technology Instrument Co., Ltd., Shanghai, China), and nuclear magnetic resonance was conducted on an AVANCE III HD 500 M (Bruker technology Co., Ltd., Billerica, MA, USA, DMSO-*d*_6_ solvents as internal standard). FT-IR spectra (Shanghai Aiyitong Network Technology Co., Ltd., Shanghai China) the samples were performed between 400 and 4000 cm^−1^ from KBr pellets. X-ray analysis (Analysis Yingzhou Technology (Shanghai) Co., Ltd., Shanghai, China) the samples was recorded on a Bruker APEX II area detector diffractometer at 296(2) K with a graphite-monochromatic MoKα radiation (λ = 0.71073 Å). The pH measurements were made with a Sartorius PB-10 digital pH meter. ESI-MS spectra were recorded by Thermo Scientific (Waltham, MA, USA) Q Exactive Orbitrap mass spectrometer in MALDI-TOF mode. Flam-Atomic Absorption Spectrometry (FAAS) was performed (Thermo Fisher Scientific, Waltham, MA, USA).

### 2.3. Synthesis of ***FP2***

**Scheme 1:**
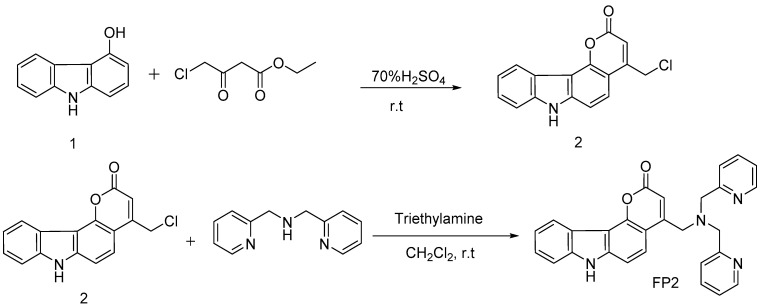
The synthetic route for preparation of compounds **FP2**.

Based on the excellent physical, chemical, and optical properties of carbazole derivatives, in this work, a novel fluorescent molecular probe **FP2** with strong blue fluorescence and coumarin as chromophore was designed and synthesized from 4-hydroxycarbazole, Ethyl 4-chloroacetoacetate and Bis (pyridin-2-yl methyl) amine (DPA) as raw materials. The probe was used to quantitatively detect the content of Zn^2+^. The synthetic route of the target compound **FP2** is outlined in Scheme 1.

#### 2.3.1. Synthesis of 4-(Chloromethyl) Pyrano[3,2-c] Carbazol-2(7H)-one (**2**)

4-hydroxycarbazole (0.57 g, 3.1 mmol) and Ethyl 4-chloroacetoacetate (0.76 g, 4.6 mmol) were dissolved in 20 mL H_2_SO_4_ (70%) in a 100 mL round bottom flask, and stirred at 25 °C under N_2_ for 5 min. The color of the solution changed to canary yellow with a black solid, and then turned to pale green after 24 h stirred. The end of the reaction was determined by TLC. Then, the mixture was poured into cool water for 20 min. The chartreuse solid was precipitated. Followed by filtration, washed with distilled water (3 × 50 mL) and anhydrous methanol (3 × 50 mL), and drying the filter cake in a vacuum drying oven, a pale-yellow solid compound **2** (0.37 g, 75% yield) was obtained: m.p. 214.1~215.3 °C; IR (KBr)ν: 3346.95, 1701.26, 1635.55, 1602.43, 1488.93, 1444.30 cm^−1^. ^1^H NMR (400 MHz, DMSO-*d*_6_) δ 12.00 (s, 1H), 8.33 (d, *J* = 7.7 Hz, 1H), 7.85 (d, *J* = 8.8 Hz, 1H), 7.63 (d, *J* = 8.2 Hz, 1H), 7.52 (dd, *J* = 17.7, 8.3 Hz, 2H), 7.35 (t, *J* = 7.5 Hz, 1H), 6.59 (s, 1H), 5.12 (s, 2H). ^13^C NMR (126 MHz, DMSO-*d*_6_) δ 186.71, 159.95, 153.36, 152.05, 141.60, 139.42, 132.99, 126.10, 124.19, 122.09, 120.19, 111.73, 111.54, 110.45, 109.36, 108.24, 99.41. HRMS (ESI) *m*/*z*: calcd for C_16_H_10_ClNO_2_ [M − H]^−^ 282.0327, found 283.0327.

#### 2.3.2. Synthesis of 4-((Bis(pyridin-2-ylmethyl) amino) methyl) Pyrano[3,2-c] Carbazol-2(7H)-one (**FP2**)

Compound **2** (0.32 g, 2 mmol) was dissolved in 10 mL anhydrous dichloromethane in a 50 mL round bottom flask. DPA (0.4 g, 2 mmol) and Triethylamine (0.61 g, 6 mmol) were added and stirred at 25 °C under N_2_. The solution was sandy beige at the beginning, obtained a tan solution. The reaction was monitored by TLC analysis until it was completed. Then, the mixture was followed by silica gel column chromatography (eluent: petroleum ether:dichloromethane:ammonia solution = 30:16:1) to obtain the **FP2** as yellow solid (0.16 g, 17.6% yield): m.p.187.8~188.8 °C; IR (KBr)ν: 3324.63, 2920.00, 1707.78, 1636.98, 1594.74, 1475.39 cm^−1^; ^1^H NMR (400 MHz, DMSO-*d*_6_) δ 12.02 (s, 1H), 8.53 (d, *J* = 4.5 Hz, 2H), 8.28 (d, *J* = 7.9 Hz, 1H), 7.84–7.73 (m, 3H), 7.60 (d, *J* = 8.1 Hz, 1H), 7.49 (dd, *J* = 13.3, 7.6 Hz, 3H), 7.42 (d, *J* = 8.6 Hz, 1H), 7.32–7.24 (m, 3H), 6.62 (s, 1H), 4.04 (s, 2H), 3.90 (s, 4H). ^13^C NMR (126 MHz, DMSO-*d*_6_) δ 173.21, 166.80, 159.94, 152.03, 150.15, 142.44, 139.44, 131.63, 131.41, 128.57, 126.07, 122.09, 120.28, 120.17, 111.53, 110.44, 109.46, 108.23, 71.04, 41.69. HRMS (ESI) *m*/*z*: calcd for C_28_H_22_N_4_O_2_ [M + H]^+^ 447.1821, found 446.1815.

### 2.4. Structure Determination

The crystal structure of compound **FP2** was determined by single-crystal X-ray diffraction. Reflection data were collected at room temperature on a Bruker APEX II area detector diffractometer equipped with a graphite-monochromatic MoKα radiation (λ = 0.71073 Å) at 296(2) K with ω-2θ scan mode. Empirical adsorption corrections were applied to all data using SADABS, version 2.10. The structures were solved by direct methods and refined by full-matrix least squares on F2 using SHELXTL 97 software, version 6.1. All non-hydrogen atoms were located by direct methods and subsequent difference Fourier syntheses. The hydrogen atoms bound to carbon were located by geometrical calculations, and their positions and thermal parameters were fixed during the structure refinement of the compound **FP2**. Crystallographic data and pertinent information are provided in Table 1. The selected bond lengths and selected bond angles are presented in Table 2.

### 2.5. Preparation of Probe ***FP2*** and Analytes

Compound **FP2** was dissolved with Dimethyl Sulfoxydum (DMSO, 1 mM) as a stock solution; CuCl_2_, MnCl_4_·4H_2_O, MgCl_2_, NaCl, KCl, CdCl_2_·2.5H_2_O, NiCl_2_·6H_2_O, ZnCl_2_, FeCl_3_·6H_2_O, SnCl_2_·2.5H_2_O, CaCl_2_·H_2_O, BaCl_2_·2H_2_O and CoCl_2_·6H_2_O were dissolved in distilled water to obtain 10 mM aqueous solutions.

### 2.6. Preparation of Water Extract of Tea as Sample

An amount of 1.0 g tea was added into 50 mL boiled distilled water for 10 min, the tea leaves were filtered, and infusions were combined for future use. Because of the interference of Cu^2+^, using a suitable saturated Na_2_S_2_O_3_ as a screening agent, the sample was added to **FP2**, and the fluorescence signals at 367 nm in the sample were recorded.

### 2.7. The Influence of Metal Ion Types on Probe Fluorescence

The concentration of fluorescent molecule probe **FP2** in the buffer system was 10 μM. CuCl_2_, MnCl_4_·4H_2_O, MgCl_2_, NaCl, KCl, CdCl_2_·2.5H_2_O, NiCl_2_·6H_2_O, ZnCl_2_, FeCl_3_·6H_2_O, SnCl_2_·2.5H_2_O, CaCl_2_·H_2_O, BaCl_2_·2H_2_O, and CoCl_2_·6H_2_O were added separately to achieve a concentration of 10 equiv in the system.

### 2.8. The Procedures of Zn^2+^ Determination and Sample Analysis

The preparation of the test system: 90 μL **FP2** and the ion solutions were added to HEPES buffer solution (25 mM, C_2_H_5_OH/H_2_O = 1:1, *v*/*v*, pH = 7), and then the solution was supplemented to 10 mL in the cuvette by adding buffer solution. After mixing, the fluorescent signals were recorded. The fluorescence spectrophotometer parameters of **FP2** measured in the preliminary experiment are as follows.

Fluorescence spectrophotometer parameters, excitation wavelength: 367 nm; emission wavelength: 460 nm; slide width: 5 nm/10 nm.

### 2.9. Verification of Analytical Methods

In HEPES buffer solution (25 mM, C_2_H_5_OH/H_2_O = 1:1, *v*/*v*, pH = 7), **FP2** (10 μM) was titrated with Zn^2+^ with gradient concentration of 0~10 μM, and a linear regression curve was drawn according to the change in the fluorescence spectrum. The results show that under these conditions, the concentration of Zn^2+^ has a good linear relationship in the range of 0~10 μM, and the correlation coefficient R^2^ > 0.99. Then, according to the concentration corresponding to the signal-to-noise ratio, the limit of detection LOD (S/N = 3) of **FP2** for Zn^2+^ was calculated.

### 2.10. Quantum Chemical Calculation

All quantum chemical calculations were conducted using the NWChem, version 7.0.2 [54]. Both geometry optimizations and Gibbs free energy calculations at 298 K were carried out using the hybrid density functional B3LYP method with a 6–31 + G(d,p) basis set [55,56,57]. The solvent effects were considered in all calculations using the integral equation formalism model (IEFPCM) [58]. Additionally, the molecular orbitals were analyzed and visualized using Multiwfn, version 3.8 and VMD software, version 1.9.4 [59,60].

## 3. Results and Discussion

### 3.1. Crystal Structure of ***FP2***

**Figure 1:**
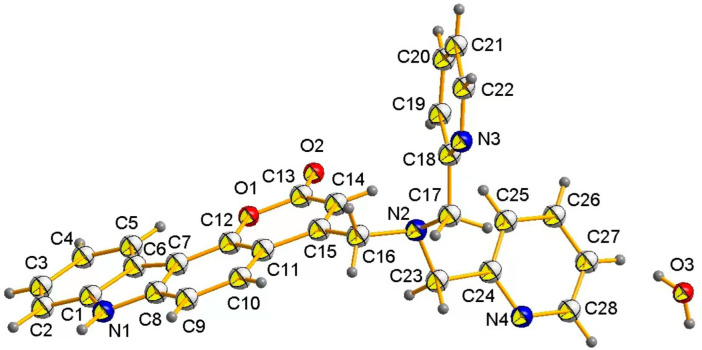
Crystal structure of compound **FP2** with 30% thermal ellipsoids.

Compound **FP2** crystallizes in the Monoclinic space group P2_1_/c. There are 4 crystallographic independent molecules in the asymmetric unit (only one of them is shown in Figure 1). The molecule of compound **FP2** consists of one water molecule and one 4-((bis (pyridin-2-ylmethyl) amino) methyl) pyrano[3,2-c] carbazol-2(7H)-one. The bond lengths and angles within the title structure are very similar to those given in the literature for 4-Methyl-2H,7H-2-oxopyrano(3,2-c) carbazole [61].

The atoms of the pyridin ring part and pyrano[3,2-c] carbazol-2 (7H)-one part is approximately planar, and the dihedral angle between the pyridin ring and the pyrano[3,2-c] carbazol-2(7H)-one ring is 62.116(105)° and 74.019(86)°. The torsion angles of C14-C15-C16-N2, C15-C16-N2-C17, C16-N2-C17-C18, C15-C16-N2-C23 and C16-N2-C23-C24 are −19.844(348)°, 158.351(213)°, −83.572(262)°, −77.921(256)° and 162.410(208)°, respectively.

### 3.2. Fluorescence Spectrum Properties of ***FP2***

**Figure 2:**
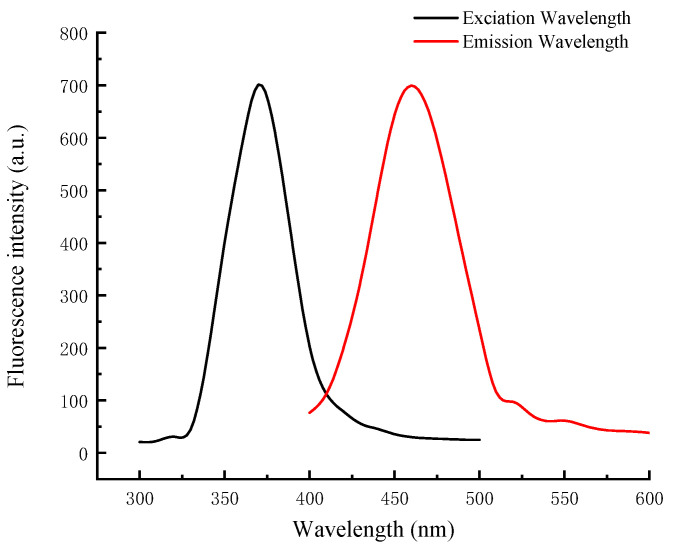
Fluorescence spectra of **FP2**.

In order to study the fluorescence signals of **FP2** and ions, we first measured the fluorescence spectral characteristics of **FP2**. As shown in Figure 2, the excitation wavelength and emission wavelength of **FP2** are 367.00 nm and 460 nm, respectively.

### 3.3. Sensing Property of Probe ***FP2*** Towards Zn^2+^

In order to realize the high-sensitivity detection of Zn^2+^ with **FP2** as a fluorescent probe, we studied the optimum media pH and reaction time that affected the detection sensitivity.

**Figure 3:**
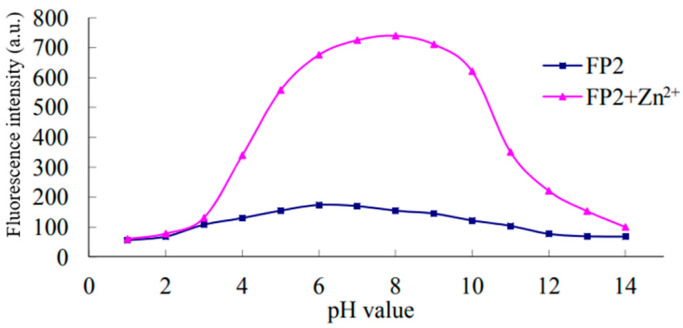
The effect of pH value of fluorescence intensity for **FP2** (10 μM) in HEPES buffer solution (25 mM, C_2_H_5_OH/H_2_O = 1:1, *v*/*v*) in the absence and presence of Zn^2+^ (10 equivalent). (λ_ex_ = 367 nm, Slit: 5 nm/10 nm).

As we can see from Figure 3, the fluorescence change in **FP2** alone was not highly dependent on pH, which means that the chemical structure and fluorescence property of **FP2** are stable over a wide range from pH 1~14. However, in the presence of Zn^2+^, the fluorescence intensity increased with the increase in pH value, and reached the maximum at pH = 8, reaching 740 a.U, and then weakened in the range from pH 8~14, which indicated that the optimum pH value for detecting Zn^2+^ was pH 7~9.

**Figure 4:**
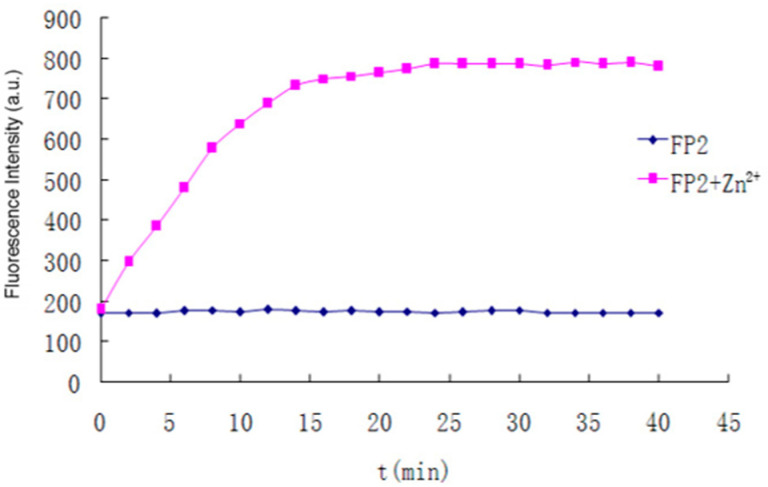
Time-dependent fluorescence intensity changes for **FP2** (10 μM) at 468nm in the presence of Zn^2+^ (10 μM/10 equiv.) in HEPES buffer solution (25 mM, C_2_H_5_OH/H_2_O = 1:1, *v*/*v*, pH = 7.0). (λ_ex_ = 367 nm, Slit: 5 nm/10 nm).

In order to ensure the accuracy of the result, the influence of reaction time was also measured. As we can see from Figure 4, the fluorescence intensity was stable and low all the time when **FP2** was alone in the HEPES buffer system. After Zn^2+^ was added, the fluorescence intensity increased shapely before the first 15 min, and remained stable after 24 min, which showed that the reaction time should be longer than 24 min.

**Figure 5:**
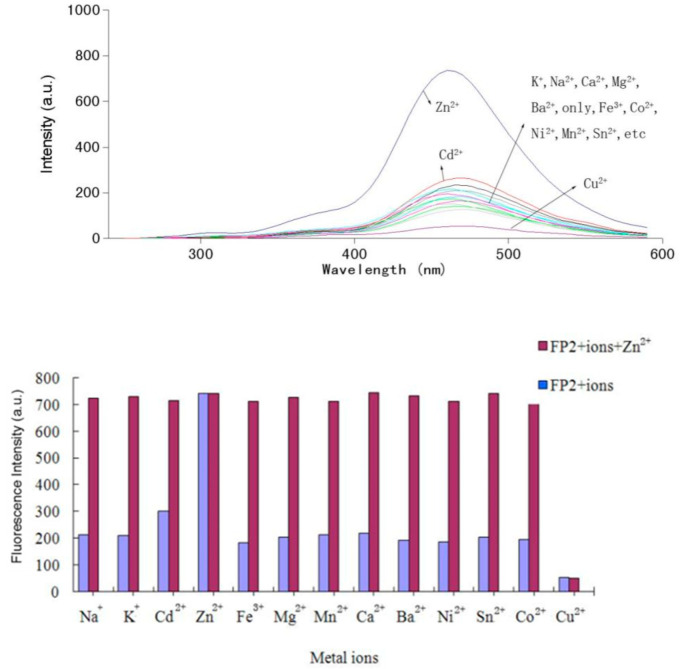
Fluorescence response of **FP2** (10 μM) to various meta ions and Specific recognition in HEPES buffer solution (25 mM, C_2_H_5_OH/H_2_O = 1:1, *v*/*v*, pH 7.0). (λ_ex_ = 367 nm, Slit: 5 nm/10 nm).

**Figure 6:**
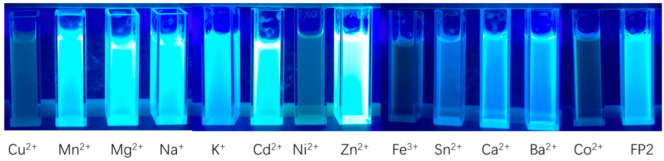
The photos of **FP2** (10 μM) to various metal ions in HEPES buffer solution (25 mM, C_2_H_5_OH/H_2_O = 1:1, *v*/*v*, pH 7.0).

For an ideal sensor, selectivity is an important parameter, which can indicate a particular kind in a complex system. Under the optimum conditions, several common metal ions were studied. The results are shown in Figure 5. Obviously, in the presence of cations without Zn^2+^, the fluorescence is only slightly enhanced, while in the presence of cations without Cu^2+^, the fluorescence is quenched, which is the same as reported results, which may be due to the inherent quenching characteristics of these ions combined with the molecular probe. Therefore, the fluorescent probe **FP2** has high selectivity to Zn^2+^ and Cu^2+^. The bright fluorescence could be observed under a 365nm UV lamp, as shown in Figure 6. Obviously, we can see that zinc ions can enhance the fluorescence of **FP2** [62,63,64,65,66].

**Figure 7:**
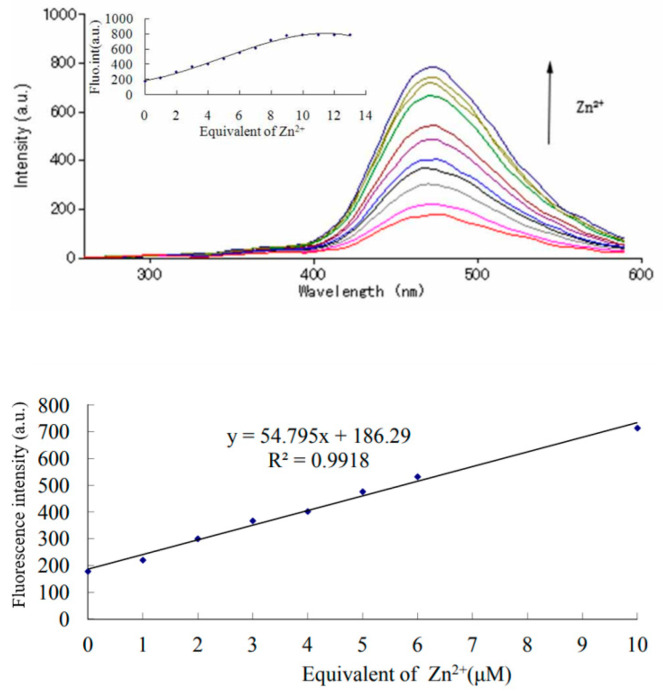
Fluorescence spectra of **FP2** (10 μM) in HEPES buffer solution (25 mM, C_2_H_5_OH/H_2_O = 1:1, *v*/*v*, pH 7.0) in the presence of different concentrations of Zn^2+^ (0~10 μM) equation of linear regression. (λ_ex_ = 367 nm, Slit: 5 nm/10 nm).

The spectroscopic properties and the metal-binding behavior of the newly synthesized probe were measured in HEPES buffer solution (25 mM, C_2_H_5_OH/H_2_O = 1:1, *v*/*v*, pH 7). The results are shown in Figure 7. The results show that the fluorescence intensity of FP2-Zn^2+^ at 469 nm increased with the increase of Zn^2+^ concentration, and the maximum fluorescence intensity was 180 a.u. In the HEPES buffer system, the fluorescence quantum yield was 0.218 when **FP2** was used alone, and the fluorescence intensity was gradually enhanced with the continuous addition of Zn^2+^. When the concentration of Zn^2+^ reached 10 μM, the fluorescence intensity reached the maximum of 732 a.u. A standard curve for the detection of Zn^2+^ was obtained and shown in Figure 7; we can see that when the equivalent of Zn^2+^ from 0~10 μM presented a good linear relationship with a correlation coefficient of R^2^ = 0.9918, and the fluorescence quantum yield was 0.69, increased by 3.2-fold. Thus, the equivalent of Zn^2+^ in the sample can be determined by using the following straight-line equation: y = 54.795x + 188.29 (y is the fluorescence intensity, x is the concentration of Zn^2+^), which indicates that **FP2** has a fine quantitative detection for Zn^2+^ in the range of 0~10 μM in HEPES buffer system. Finally, according to the concentration corresponding to the signal-to-noise ratio, the LOD (S/N = 3) of **FP2** for Zn^2+^ was calculated. The value was found to be 0.0065 μmol/L for Zn^2+^, which was lower than the previously reported detection limit of Zn^2+^.

To further demonstrate the advancement of the established method, the proposed method was compared with previously reported methods [67,68,69,70,71,72]. As shown in Table 3, this established method provided the lowest detection limit, and higher sensitivity compared with other existing methods for zinc ion. In this study, the detection limit of **FP2** fluorescence was 0.0065 μmol/L.

### 3.4. The Binding Mode of Zn^2+^ & ***FP2***

**Figure 8:**
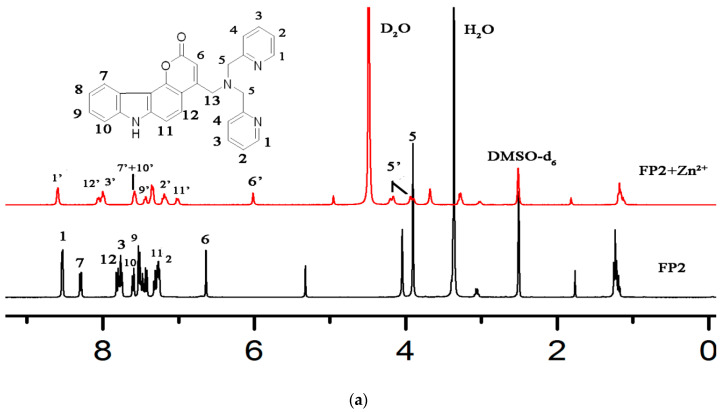

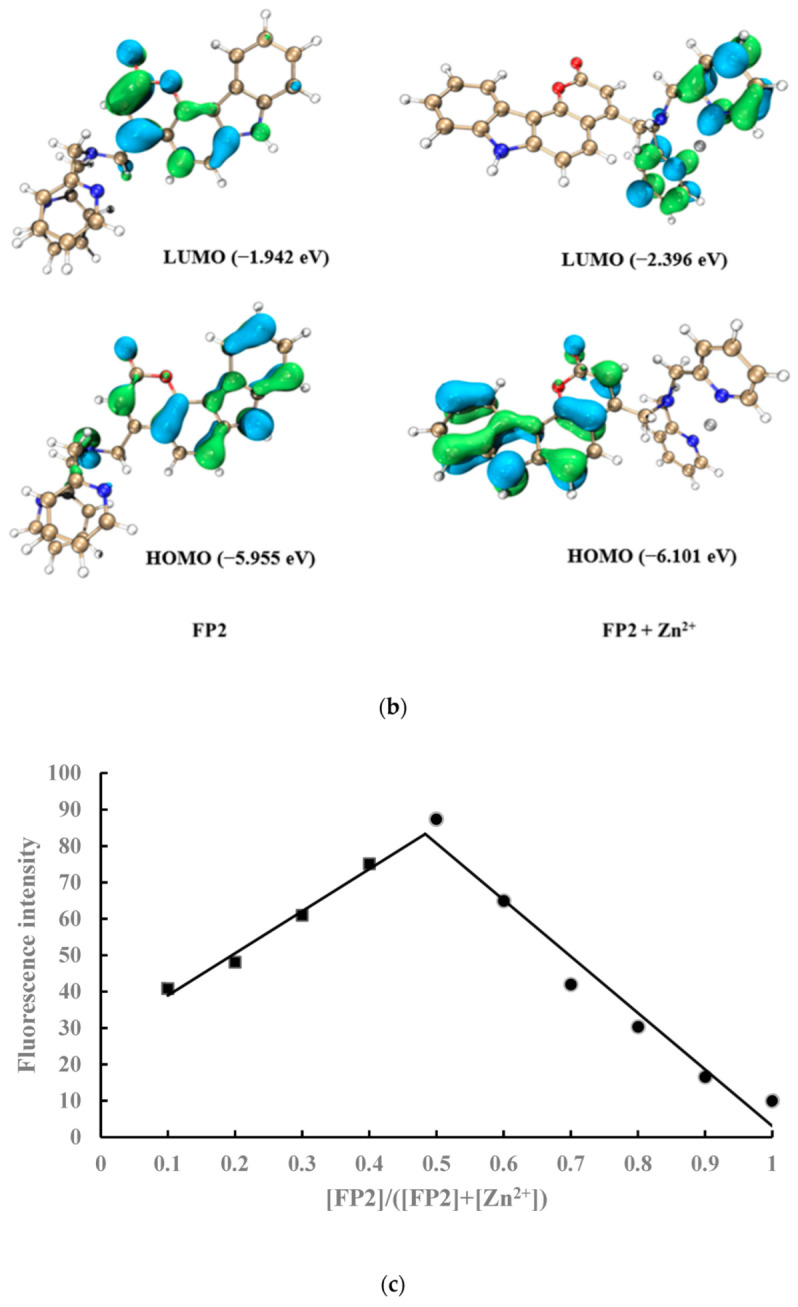
(**a**) ^1^H NMR spectra of **FP2** (1 mM) in the absence and presence of Zn^2+^ (1 equiv) in DMSO-*d*_6_; (**b**) the HOMOs and LUMOs of **FP2** and FP2 + Zn^2+^ (The isosurface values are set to 0.04 a.u.); (**c**) the job plot of **FP2** with Zn^2+^ in HEPES buffer solution (25 mM, C_2_H_5_OH/H_2_O = 1:1, *v*/*v*, pH 7.0) (λex = 367 nm, Slit: 5 nm/10 nm).

In order to further study the binding mode of Zn^2+^ and **FP2**, ^1^HNMR titration experiments were carried out. ^1^H NMR spectra studies were undertaken using DMSO-*d*_6_ as the solvent. Addition of Zn^2+^ to solutions of **FP2** resulted in the formation of a new set of ^1^H NMR signals in addition to the set arising from free **FP2**. We can clearly see these changes in Figure 8a. The protons in the ortho position of the pyridine were shifted from about 8.44 ppm to 8.55 ppm due to the addition of Zn^2+^. A similar shift was observed at the 2-, 3- and 11-positions of pyridine; the H atoms at the 5-position of DPA were split and shifted; and the 6-position hydrogen atom moved obviously from 6.6 ppm to 6.0 ppm. In addition, through the calculation of density functional theory, a further structural explanation of this binding mode was obtained. As shown in Figure 8b, after combining with Zn^2+^, the energy gap of HOMO-LUMO decreased by about 0.309 eV, indicating that **FP2** became more stable after combining with Zn^2+^. According to the job plot of **FP2** with Zn^2+^ in the HEPES buffer solution, the binding mode can also be verified. As can be seen from Figure 8c, when **FP2**/(**FP2** + **Zn^2+^**) = 0.5, the fluorescence signal intensity reaches the maximum, indicating that **FP2** and Zn^2+^ are combined in a 1:1 manner.

## 4. Determination of Zn^2+^ in the Water Extract of Tea

In order to ensure this probe can be applied in real life, we used the water extract of tea as the sample to detect the concentration of Zn^2+^ by the method of flame atomic absorption spectrometry (FAAS) and **FP2** and the result was shown in Table 4 and Table 5. As can be seen from Table 4, we can see that the results were similar, and the recoveries were 97~101%. From Table 5, we can see that the Relative Standard Deviation (RSD) and Coefficient of Variation (CV) were 1.51% and 3.5% lower than using the method of flame atomic absorption spectrometry (FAAS), which indicates that the method of using **FP2** was more accurate in detecting the Zn^2+^ in the water extract of tea.

## 5. Conclusions

Based on the excellent physical, chemical, and optical properties of carbazole derivatives, in this work, a novel fluorescent molecular probe, **FP2**, with strong blue fluorescence and coumarin as chromophore, was designed and synthesized from 4-hydroxycarbazole, Ethyl 4-chloroacetoacetate and Bis (pyridin-2-yl methyl) amine (DPA) as raw materials. The probe was used to quantitatively detect the content of Zn^2+^. The structure of **FP2** was characterized by ^1^HNMR, HRMS, and X-ray diffraction. In the HEPES buffer solution, **FP2** was responsive to Zn^2+^ and greatly enhanced. The pH value and reaction time were investigated, and the optimum reaction conditions were determined as follows: the pH was 7~9 and the reaction time was longer than 24 min. Under the optimized conditions, the concentration of **FP2** and Zn^2+^ showed a good linear relationship in the range of 0~10 μM, and the LOD was 0.0065 μmol/L. In addition, through the ^1^H NMR titration experiment, density functional theory calculation, and the job plot of **FP2** with Zn^2+^ in the HEPES buffer solution, the binding mode of **FP2** and Zn^2+^ was explained. Finally, FAAS and **FP2** were used to detect the content of Zn^2+^ in the water extract of tea. The results showed that the **FP2** method is more accurate than the FAAS method, which shows that the method described in this work could be used to detect the content of Zn^2+^ in practical samples and verify the practicability of this method.

## Figures and Tables

**Table 1:** The crystallographic data of compound **FP2**.

Empirical Formula	C_28_H_24_N_4_O_3_	V (Å^3^)	2388.0(8)
Formula weight	464.51	Z	4
Temperature (K)	296(2)	Dc (g/cm^3^)	1.292
Wavelength (Å)	0.71073	Absorption coefficient (mm^−1^)	0.086
Crystal system	Monoclinic	F(000)	976
Space group	P2_1_/c	Crystal size (mm)	0.220 × 0.200 × 0.160
*a* (Å)	16.762(3)	*Θ* range for data collection (°)	2.520–25.496
*b* (Å)	8.8948(18)	Reflections collected	16,936
*c* (Å)	16.606(3)	Independent reflection	4440 [R(int) = 0.0450]
*α* (°)	90	Goodness-of-fit on *F*^2^	1.014
*β* (°)	105.301(3)	Final *R* indices [*I* > 2*σ*(*I*)]	*R*_1_ = 0.0523, *wR*_2_ = 0.1455
*γ* (°)	90	*R* indices (all data)	*R*_1_ = 0.0945, *wR*_2_ = 0.1812

*R*_1_ = Σ║*F_o_*│ − │*F_c_*║/Σ│*F_o_*│; *wR*_2_ = [Σ*w*(*F_o_*^2^ − *F_c_*^2^)_2_/Σ*w*(*F_o_*^2^)^2^]^1/2^.

**Table 2:** The bond length [Å] and bond angles [°] of compound **FP2**.

Bond	[Å]	Bond	[Å]	Bond	[Å]
C(1)-C(6)	1.390(4)	C(10)-C(11)	1.408(3)	C(18)-C(19)	1.365(4)
C(1)-C(2)	1.394(4)	C(11)-C(12)	1.398(3)	C(19)-C(20)	1.381(4)
C(1)-N(1)	1.396(4)	C(11)-C(15)	1.451(3)	C(20)-C(21)	1.351(4)
C(2)-C(3)	1.370(5)	C(12)-O(1)	1.366(3)	C(21)-C(22)	1.343(5)
C(3)-C(4)	1.361(5)	C(13)-O(2)	1.208(3)	C(22)-N(3)	1.336(4)
C(4)-C(5)	1.378(4)	C(13)-O(1)	1.358(3)	C(23)-N(2)	1.464(3)
C(5)-C(6)	1.392(4)	C(13)-C(14)	1.432(4)	C(23)-C(24)	1.500(3)
C(6)-C(7)	1.458(4)	C(14)-C(15)	1.338(3)	C(24)-N(4)	1.336(3)
C(7)-C(12)	1.379(3)	C(15)-C(16)	1.509(3)	C(24)-C(25)	1.377(4)
C(7)-C(8)	1.391(4)	C(16)-N(2)	1.457(3)	C(25)-C(26)	1.374(4)
C(8)-N(1)	1.389(4)	C(17)-N(2)	1.459(3)	C(26)-C(27)	1.362(4)
C(8)-C(9)	1.391(4)	C(17)-C(18)	1.510(4)	C(27)-C(28)	1.366(5)
C(9)-C(10)	1.382(4)	C(18)-N(3)	1.328(3)	C(28)-N(4)	1.343(4)
**Angles**	**[°]**	**Angles**	**[°]**	**Angles**	**[°]**
C(6)-C(1)-C(2)	122.3(3)	C(12)-C(11)-C(15)	116.9(2)	C(21)-C(20)-C(19)	118.8(3)
C(6)-C(1)-N(1)	109.4(3)	C(10)-C(11)-C(15)	124.6(2)	C(22)-C(21)-C(20)	118.4(3)
C(2)-C(1)-N(1)	128.3(3)	O(1)-C(12)-C(7)	116.4(2)	N(3)-C(22)-C(21)	124.2(3)
C(3)-C(2)-C(1)	115.9(3)	O(1)-C(12)-C(11)	122.3(2)	N(2)-C(23)-C(24)	112.7(2)
C(4)-C(3)-C(2)	123.2(3)	C(7)-C(12)-C(11)	121.3(2)	N(4)-C(24)-C(25)	121.2(2)
C(3)-C(4)-C(5)	121.1(3)	O(2)-C(13)-O(1)	116.5(2)	N(4)-C(24)-C(23)	115.2(2)
C(4)-C(5)-C(6)	118.1(3)	O(2)-C(13)-C(14)	126.8(3)	C(25)-C(24)-C(23)	123.5(2)
C(1)-C(6)-C(5)	119.5(3)	O(1)-C(13)-C(14)	116.8(2)	C(26)-C(25)-C(24)	120.4(3)
C(1)-C(6)-C(7)	106.2(3)	C(15)-C(14)-C(13)	123.7(3)	C(27)-C(26)-C(25)	118.7(3)
C(5)-C(6)-C(7)	134.3(3)	C(14)-C(15)-C(11)	118.5(2)	C(26)-C(27)-C(28)	118.2(3)
C(12)-C(7)-C(8)	118.5(3)	C(14)-C(15)-C(16)	121.0(2)	N(4)-C(28)-C(27)	124.1(3)
C(12)-C(7)-C(6)	134.1(2)	C(11)-C(15)-C(16)	120.4(2)	C(8)-N(1)-C(1)	108.3(2)
C(8)-C(7)-C(6)	107.3(2)	N(2)-C(16)-C(15)	113.4(2)	C(16)-N(2)-C(17)	111.2(2)
N(1)-C(8)-C(9)	128.8(3)	N(2)-C(17)-C(18)	113.4(2)	C(16)-N(2)-C(23)	110.93(19)
N(1)-C(8)-C(7)	108.9(3)	N(3)-C(18)-C(19)	121.2(2)	C(17)-N(2)-C(23)	110.79(19)
C(9)-C(8)-C(7)	122.3(3)	N(3)-C(18)-C(17)	114.8(2)	C(18)-N(3)-C(22)	117.7(3)
C(10)-C(9)-C(8)	118.0(2)	C(19)-C(18)-C(17)	123.9(2)	C(24)-N(4)-C(28)	117.4(2)
C(9)-C(10)-C(11)	121.5(3)	C(18)-C(19)-C(20)	119.7(3)	C(13)-O(1)-C(12)	121.7(2)
C(12)-C(11)-C(10)	118.4(2)				

**Table 3:** Comparison of the proposed method and the previously reported methods.

Analytical Method	LOD (μmol/L)	Ref.
fluorescence probe technology	0.0137	[67]
fluorescence probe technology	0.0922	[68]
fluorescence probe technology	0.066	[69]
fluorescence probe technology	0.298	[70]
flame atomic absorption spectrometry	0.0765	[71]
Photoelectric colorimetry	1.2236	[72]
fluorescence probe technology	0.0065	Our work

**Table 4:** The result of the two methods of the concentration of Zn^2+^ in the water extract of tea (μg/mL).

Method	The Concentration of Zn^2+^ in the Water Extract of Tea				Standard Sample	The Concentration of Zn^2+^ After Added Standard Sample				Recovery (%)
	1	2	3	Average		1 + Ions	2 + Ions	3 + Ions	Average	
FAAS	42.5	42.7	39.5	41.6 ± 1.8	10 (Zn^2+^)	52.9	51.9	51.0	51.9 ± 1.0	98~104.8
FP2	42.6	41.9	44.8	43.1 ± 1.5	10 (Zn^2+^)	51.0	50.3	52.7	51.3 ± 1.2	97~101

**Table 5:** The comparison between the two methods from accuracy.

Method	Range (μg/mL)	$\bar{X}$	RSD (%)	CV (%)
FP2	41.9~44.8	43.1	1.51	3.5
FAAS	39.5~42.7	41.6	1.79	4.3

## Data Availability

Data are contained within the article and Supplementary Materials.

## Supplementary Materials

The following supporting information can be downloaded at: https://www.mdpi.com/article/10.3390/molecules29225454/s1, Figure S1: ^1^HNMR for FP1 in DMSO-*d*_6_; Figure S2: ^13^CNMR for FP1 in DMSO-*d*_6_; Figure S3: ^1^HNMR for **FP2** in DMSO-*d*_6_; Figure S4: ^13^CNMR for **FP2** in DMSO-*d*_6_; Figure S5: FT-IR for FP1; Figure S6: FT-IR for **FP2**; Figure S7: HRMS for FP1; Figure S8: HRMS for **FP2**.
