# Supplementary material for: Design, Synthesis and Crystal Structure of a Novel Fluorescence Probe for Zn2+ Based on Pyrano[3,2-c] Carbazole
Source: Molecules. 2024 Nov 19;29(22):5454. doi: 10.3390/molecules29225454 (PMC11597256; doi:10.3390/molecules29225454)
Supplement: Supplementary file 1 [file molecules-29-05454-s001.zip › molecules-3278405-supplementary.pdf]

Supporting Information

Design, Synthesis and Crystal Structure of a Novel Fluorescence Probe for Zn^{2+} Based on Pyrano[3,2-c] Carbazole

Zi-Yin Xie [1], Qing-Wen Fang[2], Shu-Zhen Xiao[1], Jie Wang [1], Ping Lin [3], Chun-Mei Guo [4], Hui-Hua Cao[5], Zhong-Ping Yin [3], Li-Hong Dong[6]*, Da-Yong Peng[1]**

Table of contents

1.NMR spectra for probe characterization..1
2. FT-IR spectra for probe characterization 3
3.HRMS spectra for probe characterization..4

1.NMR spectra for probe characterization

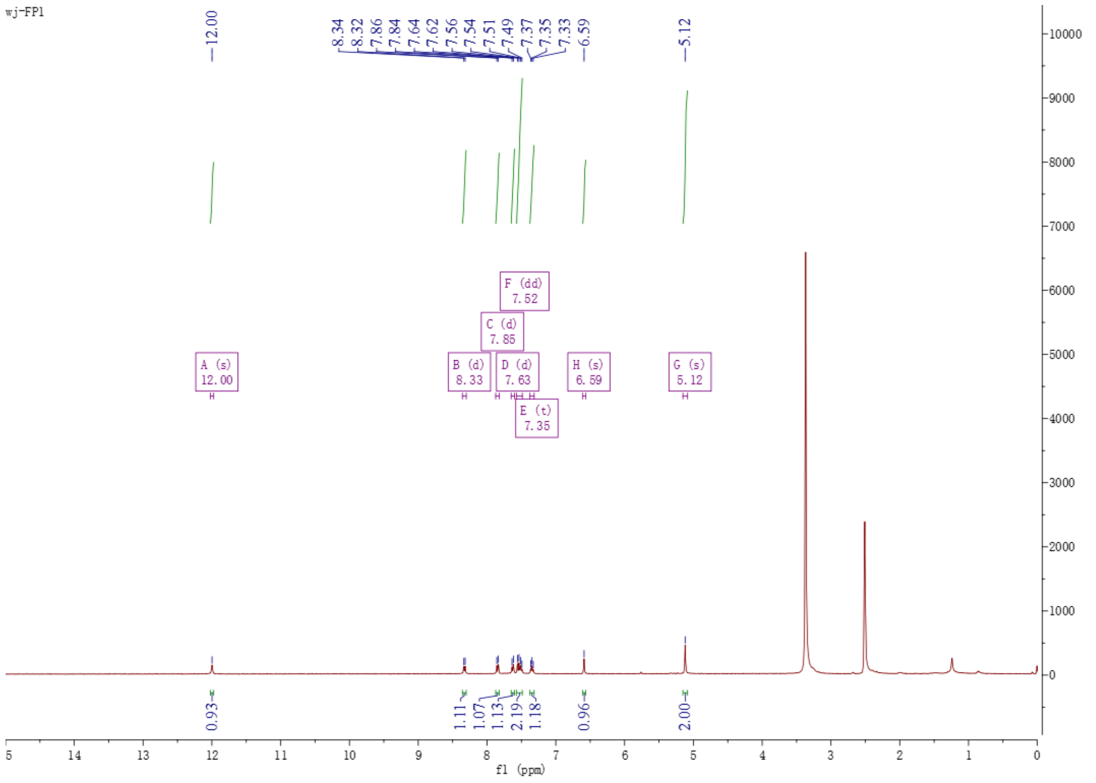

Figure S1. ^{1}HNMR for FP1 in DMSO-d_6

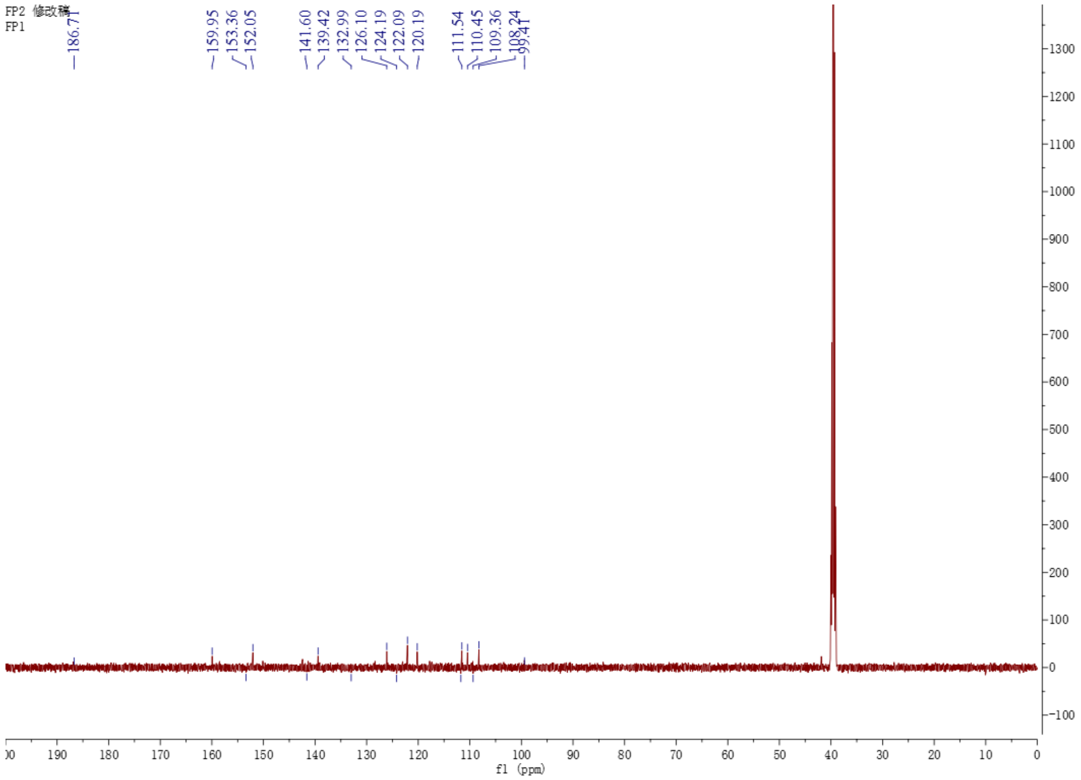

Figure S2 ^{13}CNMR for FP1 in DMSO-d_6

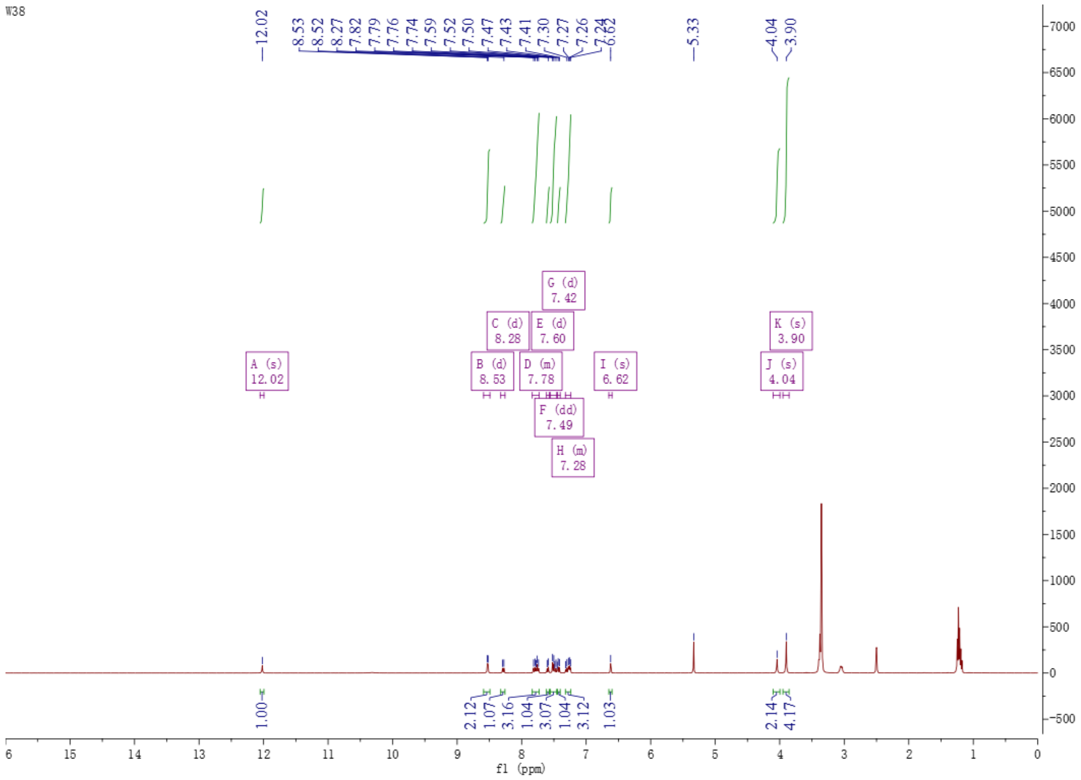

Figure S3 ^{1}HNMR for FP2 in DMSO-d_6

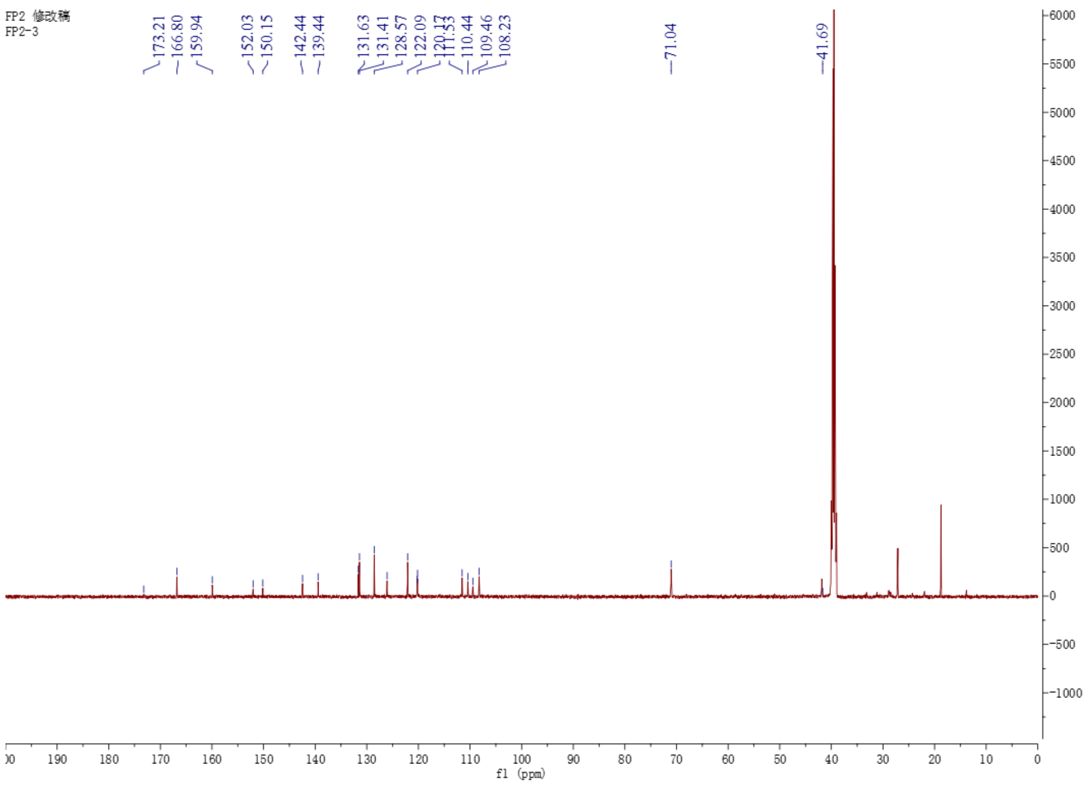

Figure S4 ^{13}CNMR for FP2 in DMSO-d_6

2. FT-IR spectra for probe characterization

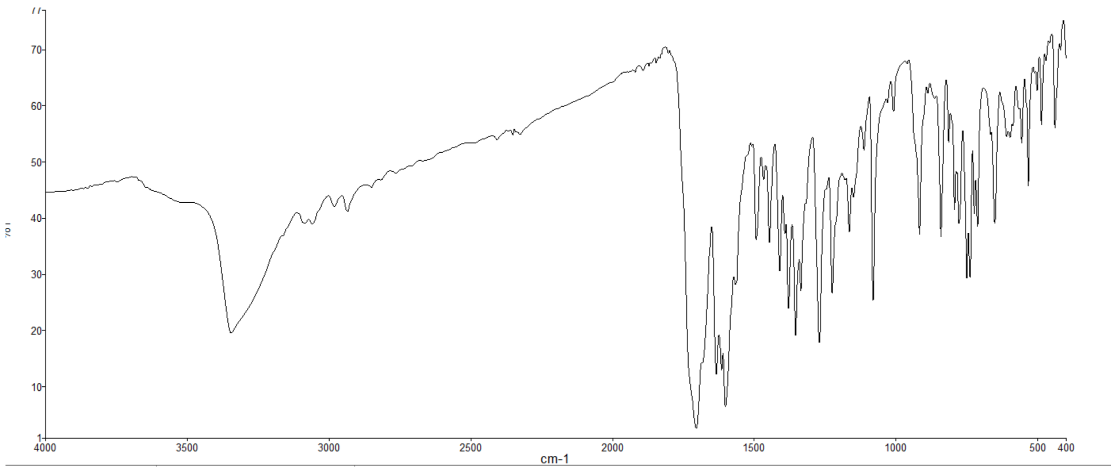

Figure S5 FT-IR for FP1

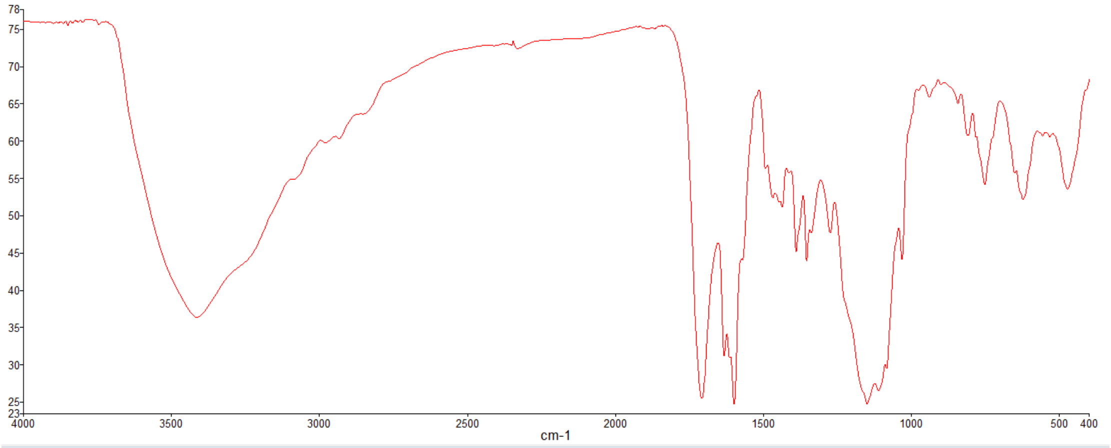

Figure S6 FT-IR for FP2

3.HRMS spectra for probe characterization

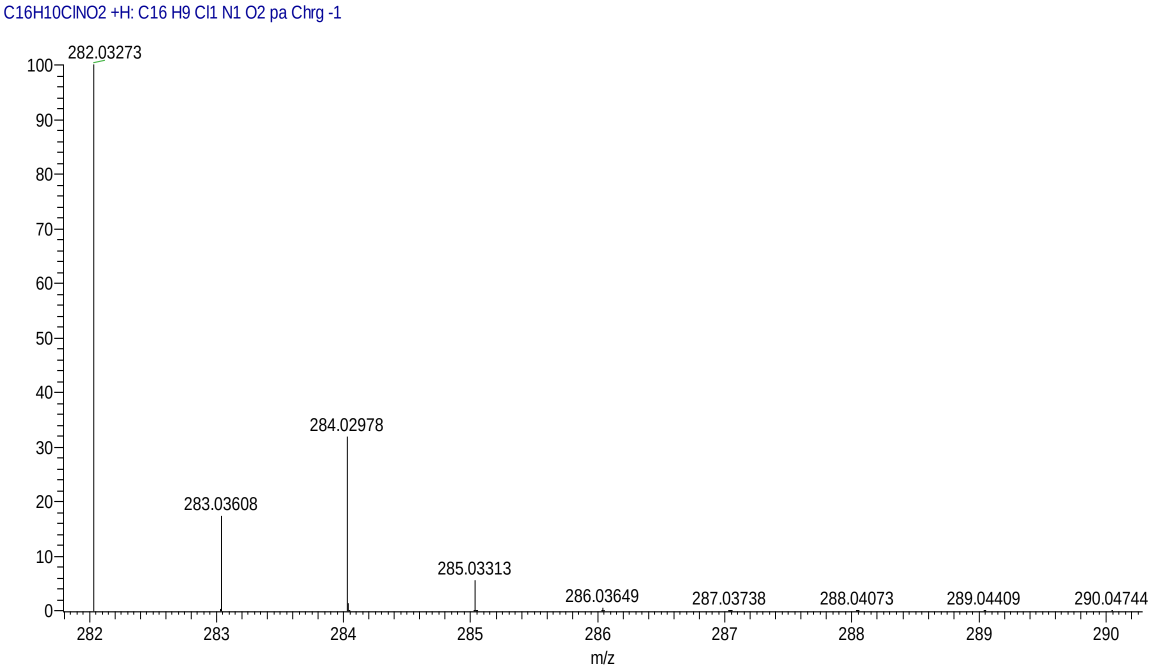

Figure S7 HRMS for FP1

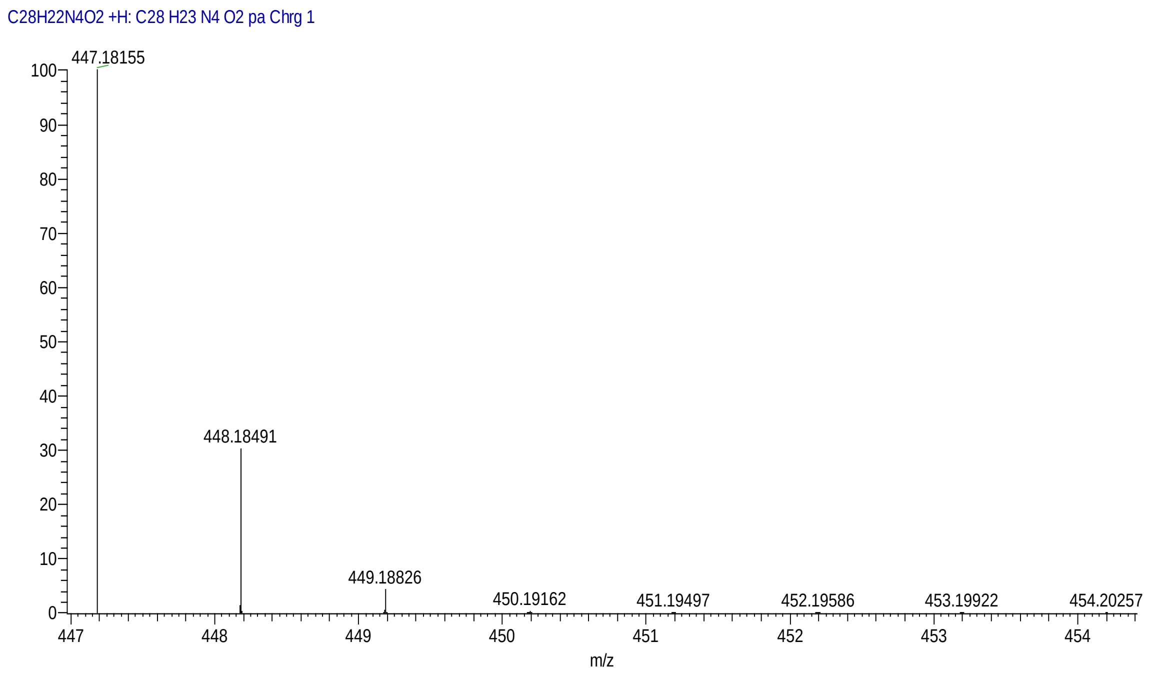

Figure S8 HRMS for FP2
